# Dental Education

**Published:** 1880-09

**Authors:** Geo. H. Perine

**Affiliations:** New York


ARTICLE III.

### DENTAL EDUCATION.

BY DR. GEO. H. PERINE, NEW YORK.

It is a well authenticated fact that dentistry has, within the past few years made rapid strides of advancement—that its improvements have been many and great. In fact it has, in this respect, far exceeded any other specialty of medicine to which it is closely related, or of which, we may say it constitutes an important branch. The subject of a closer union of medicine and dentistry, has for years, been agitated, yet it must be acknowledged, the result has not proved fully satisfactory, owing no doubt to the evident lack of interest displayed by prominent men in both branches of the science, whose co-operation in the direction referred to, would doubtless lead to the most satisfactory and valuable result. The writer holds, that dentistry is as much a branch of the medical science as is surgery or any other of its acknowledged specialties, and therefore strongly advocates the establishment of dental chairs in our medical colleges. True, but partial step in this direction has in a few instances been taken; but why not make the movement general and more decided in its character? For allowing dentistry to be a medical specialty—which is an undeniable fact, it must be conceded that something beyond a mere mechanical knowledge is needed. There are those styling themselves dentists—good mechanics—holding diplomas which have been issued by dental colleges, who have little or no knowledge of oral surgery. To fully meet the requirements of our specialty, a perception extended beyond the mere method of extracting and filling teeth, and the construction and adjustment of artificial dentures is necessary. A perfect knowledge of surgery is in fact indispensible. An acquaintance with pathology, physiology, anatomy and chemistry needful to enable the practitioner to properly treat the disorders of the buccal cavity, and accidents to the jaws? Many of the diseases which come under the eye of the dentist have their origin in a part of the body remote from the head, and the knowledge which enables the dentist to detect this fact is often a great assistance to him in his mode of treatment.

It is hardly necessary to touch upon the care to be exercised in the use of an anesthetic, where delicate surgical operations are to be performed, and the treatment of the mouths of females during certain conditions. In a word every dentist should possess that knowledge which the degree of Doctor of medicine is supposed to imply and which should embrace dentistry, as well as the other medical spcialties and which will be imparted only when medicine and dentistry are united.

I ask that the leading members of our specialty and dental societies generally, give this matter serious consideration, for I feel assured that all that is required to bring about the establishment of a class of dental surgery in each of the State Medical Institutions, is the co-operation of the dental press and a decided advocation of the matter by leading members of our specialty.

